# The Complex Relationship of Extracorporeal Membrane Oxygenation and Acute Kidney Injury: Causation or Association?

**DOI:** 10.1155/2016/1094296

**Published:** 2016-02-24

**Authors:** Daniel J. Kilburn, Kiran Shekar, John F. Fraser

**Affiliations:** ^1^Critical Care Research Group, The Prince Charles Hospital, 627 Rode Road, Chermside, Brisbane, QLD 4032, Australia; ^2^School of Medicine, The University of Queensland, Brisbane, Australia; ^3^Royal Brisbane and Women's Hospital, Brisbane, Australia

## Abstract

Extracorporeal membrane oxygenation (ECMO) is a modified cardiopulmonary bypass (CPB) circuit capable of providing prolonged cardiorespiratory support. Recent advancement in ECMO technology has resulted in increased utilisation and clinical application. It can be used as a bridge-to-recovery, bridge-to-bridge, bridge-to-transplant, or bridge-to-decision. ECMO can restitute physiology in critically ill patients, which may minimise the risk of progressive multiorgan dysfunction. Alternatively, iatrogenic complications of ECMO clearly contribute to worse outcomes. These factors affect the risk : benefit ratio of ECMO which ultimately influence commencement/timing of ECMO. The complex interplay of pre-ECMO, ECMO, and post-ECMO pathophysiological processes are responsible for the substantial increased incidence of ECMO-associated acute kidney injury (EAKI). The development of EAKI significantly contributes to morbidity and mortality; however, there is a lack of evidence defining a potential benefit or causative link between ECMO and AKI. This area warrants investigation as further research will delineate the mechanisms involved and subsequent strategies to minimise the risk of EAKI. This review summarizes the current literature of ECMO and AKI, considers the possible benefits and risks of ECMO on renal function, outlines the related pathophysiology, highlights relevant investigative tools, and ultimately suggests an approach for future research into this under investigated area of critical care.

## 1. Introduction

Extracorporeal membrane oxygenation (ECMO) is being increasingly used in patients with cardiorespiratory failure whilst even more novel applications continue to evolve. ECMO may reduce mortality in patients with cardiopulmonary failure of multiple aetiologies, although conclusive evidence is still lacking [1–5]. ECMO is not a definitive treatment; it is a temporary support system that allows organ support whilst potentially disease-modifying therapy is administered or more definitive therapies are planned [6]. The institution of salvage ECMO is often reserved for patients with a predicted mortality rate in excess of 80% [7]. Improvements in clinical application and device design with a more precise appreciation of the patient-device interface, particularly with regard to haematological changes, have improved the risk : benefit ratio of ECMO [8–10].

An increase in the scope of indications has led to the implementation of ECMO in numerous critical care settings. These include the emergency department, specialized interhospital retrieval services, cardiac catheterisation lab, and during perioperative cardiac surgical management [8]. Despite this, mortality rates range from 40% to 60% in the adult population [4–6, 9, 11, 12]. Initiation of ECMO can lead to restitution of physiology which may prevent the progression of preexisting disease or minimise harm caused by salvage therapy (i.e., frusemide infusion and/or inotropic support). Despite this, morbidity and mortality are predominantly caused by progression of preexisting disease; however iatrogenic factors contribute significantly. Thrombosis and haemorrhage are the most common serious iatrogenic complications, which result from a complex systemic response initiated by blood-surface interaction and a subsequent use of unfractionated heparin [13, 14].

Equally, acute kidney injury (AKI) is also common in patients on ECMO, with incidence as high as 70%–85% [7, 15]. AKI in conjunction with ECMO is associated with increased mortality rates of up to 80% [4, 5, 11, 16–19]. AKI is present both before and after initiation of ECMO; however clarity in regard to the timing and risk factors for major renal insult is lacking. Pharmacological therapy directed at preventing ECMO initiation is often implemented at the expense of kidney function and it is unclear whether the commencement of ECMO protects or exacerbates this. Despite the extensive literature investigating AKI in the setting of cardiopulmonary bypass (CPB), there are insufficient mechanistic and clinical data defining the link between ECMO and AKI [20]. A comprehensive understanding of this relationship and subsequent investigation of ECMO-associated AKI (EAKI) would enable clinicians to predict, treat, and ameliorate the clinical sequelae.

## 2. ECMO Technique and Perfusion Strategies

**Figure 1:**
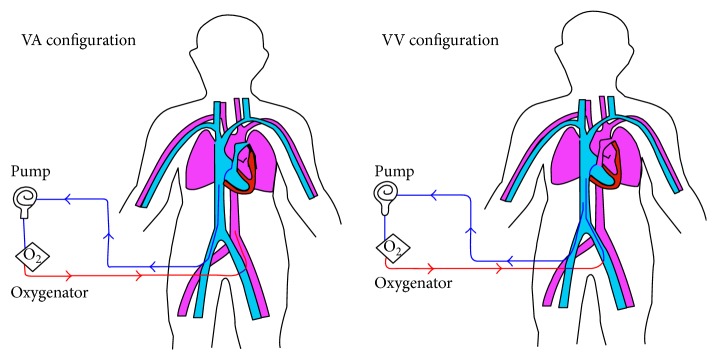
The various cannulation configurations used for ECMO. In both configurations, deoxygenated blood (blue line) is removed from the venous circulation. In venoarterial (VA) ECMO, oxygenated blood (red line) is returned to arterial circulation. Conversely, in venovenous (VV) ECMO the return is made to the venous system (reproduced with permission from Elsevier).

ECMO can be implemented as a venoarterial (VA) or venovenous (VV) configuration, determined by the clinical context (Figure 1) [8, 13]. Peripheral and central VA ECMO is reserved for patients with inadequate cardiac function and may result in a pulseless circulation, especially when there is minimal native cardiac output [1]. VV ECMO is used for patients with isolated refractory respiratory failure and does not provide direct circulatory support [8]. These fundamental differences between VA and VV ECMO may have significant implications on the development of EAKI. A comprehensive review of the different types of ECMO cannulation is beyond the scope of this paper but is addressed in other journals [1, 8].

## 3. ECMO-Associated Kidney Injury: Can Traditional Definitions Be Applied?

AKI is characterized by an abrupt loss in renal excretory function leading to the retention of nitrogenous waste products and dysregulation of extracellular volume and electrolytes [21]. The incidence of AKI in critically ill patients is increasing, complicating the clinical course of almost 60% of patients admitted to intensive care units (ICU) [22, 23]. Due to the complexity of diagnosis, various unstandardized definitions and diagnostic criteria have existed and are still present throughout the literature [24]. This has hindered the advancement of research on AKI due to confounding comparisons among studies [25, 26]. Because of this, bias in the literature examining diagnosis, incidence, and attributable mortality of AKI exists [27].

Recently, consensus on the definition of AKI by the Acute Dialysis Quality Initiative has been achieved [24]. The original RIFLE (risk, injury, failure, loss, and end stage) criteria and the revised Acute Kidney Injury Network (AKIN) criteria are now commonly used and well established throughout the literature [28, 29]. Furthermore, these definitions have been consolidated into the KDIGO Clinical Practice Guidelines for AKI Definition [24, 30]. RIFLE and AKIN have been validated in various population settings, including patients on ECMO [20, 21, 31–35].

Despite subtle differences between RIFLE and AKIN (Table 1), it is not clear whether one is superior to the other, particularly in those supported by an ECC [24, 25, 29, 31, 36]. Both have high sensitivity and specificity for diagnosing AKI [24, 25, 29, 31, 36, 37]. Furthermore, they correlate with clinical outcomes including length of hospital stay, requirement for renal replacement therapy (RRT), and overall mortality [29, 33, 38]. For ECMO, these criteria have good short-term prognostic capability and an independent mortality relationship exists for class-failure (RIFLE) and stage 3 (AKIN) (Table 2) [16, 31]. Despite this, research into the utility of RIFLE and AKIN criteria during ECMO and for the recognition of EAKI is limited and needs further investigation.

Neither RIFLE nor AKIN is perfect and a number of recognized limitations exist which may affect their efficacy in ECMO. Both utilise surrogate and delayed markers of renal impairment (i.e., serum creatinine and urine output) to diagnose and stage AKI. Serum creatinine (sCr) is an insensitive and unreliable biomarker for early detection of kidney injury and recovery [39]. Changes in sCr only occur with a >50% decline in glomerular filtration rate (GFR) and, along with urine output, can be influenced by a number of factors unrelated to renal dysfunction [39]. Early and accurate detection is important as “transient AKI” (the resolution of AKI within 72 hours of onset) is common and correlates independently with increased mortality [40]. In view of these limitations, future refinements should consider markers that are more sensitive and specific to the overall accuracy of diagnosis and early detection of renal impairment in patients on ECMO. Identifying the pathophysiological factors and physiological mechanisms that occur to the kidney in the context of ECMO may improve the accuracy and speed of diagnosis of EAKI.

## 4. Pathophysiology of ECMO-Associated Kidney Injury

It is clear that the pathophysiological features of EAKI are complex, multifactorial, and time dependant and likely to be synergistic. There is limited literature assessing the pathophysiological mechanisms of AKI and ECMO; however there is research that describes CPB-associated AKI. CPB produces a predominantly pulseless system, VA ECMO typically has limited pulsatility, and VV ECMO is always pulsatile. However, due to other similarities between CPB and ECMO, it is likely that there are at least some common underlying factors with VV and VA ECMO. Progression of preexisting multisystemic disease, pre-ECMO management, activation of proinflammatory mediators, alterations in renal macro/microvasculature, ischaemia-reperfusion, haemolysis and oxidative stress, disruption of the glycocalyx, impaired renal autoregulation, iatrogenic nephrotoxicity (i.e., antibiotics), and iatrogenic coagulation abnormalities are possible factors involved in the pathogenesis of EAKI [41]. These processes occur within the pre-ECMO, ECMO, and post-ECMO setting and may be prevented or exacerbated by ECMO initiation. Regardless, they are fundamental features leading to increased rates of AKI in the presence of an ECC [42, 43]. Ultimately, they may contribute to the development of EAKI, a subset of type 1 and type 3 cardiorenal syndrome [44].

### 4.1. Systemic Inflammatory Response

Activation of proinflammatory mediators caused by the underlying disease process, combined with the interaction of blood on the nonendothelialised ECMO interface, may initiate and amplify a systemic inflammatory response. Proinflammatory cytokines such as interleukin-1 (IL-1), IL-6, IL-8, and tumour necrosis factor-alpha (TNF-*α*) are believed to be the main contributors to this process [42, 43]. These cytokines are released from activated leukocytes in the presence of endothelial cell injury and a nonphysiological extracorporeal surface, which should represent a coordinated systemic response in patients on ECMO [40]. However, a pathologic dysregulated host response with a hyperinflammatory cytokine storm may induce alterations of the glycocalyx and microcirculatory dysfunction and induce aseptic renal inflammation and injury [40, 45–48]. This exaggerated inflammatory response may be due to an overactivation of toll-like receptors and thus an increase in the transcription of factors that regulate the expression of proinflammatory cytokines [49]. An early hyperimmune and a later immunosuppressive response leading to sepsis-associated AKI have been suggested in the literature, and relevant targeted therapies to induce renoprotection continue to emerge [41, 46, 50–53]. A time-dependant immune response and subsequent approach to treatment may also be useful in EAKI; however this has not yet been investigated.

### 4.2. Renal Macro/Microcirculatory Dysfunction

A systemic inflammatory response may also cause alterations in regional macro- and microvascular blood flow and vasomotor tone, often leading to a hyperdynamic, vasodilated state while on ECMO [52]. Similar to sepsis, this clinical phenotype manifests with tachycardia, hypotension, and an elevated cardiac output. Although difficult to substantiate in humans, due to the invasiveness of renal blood flow (RBF) monitoring, an increase in RBF in patients with this clinical phenotype on ECMO has been postulated [54, 55]. Animal models of sepsis-associated AKI have demonstrated renal hyperaemia with increased RBF and decreased renal vascular conductance (RVC) in the presence of a decreased GFR [54, 56, 57]. This suggests an innate dissociation between RBF and renal function and a possible flow/perfusion (F/Q) mismatch, highlighting the role of the renal microvasculature in the development of EAKI. Disproportionate dilatation of the efferent arteriole compared to the afferent arteriole may reduce glomerular filtration pressure, leading to a decrease in GFR. However, the presence of microcirculatory dysfunction with possible preglomerular arterial-venous (AV) or arteriolar-arteriolar (AA) renal oxygen shunting may also account for this [58–62].

Microcirculatory dysfunction is likely to play a central role in the pathogenesis of EAKI, through both direct and “organ cross talk” mechanisms [47, 63]. In pathological and nonpathological states, the autoregulation of renal microvasculature is responsible for the delicate balance of oxygen supply versus demand at the mitochondrial level [47]. An inflammatory and hypoxia-induced renal microvascular autoregulatory dysfunction with endothelial glycocalyx degradation results in precapillary sphincter relaxation and endothelial cell dysfunction [47, 48, 64, 65]. This leads to a heterogeneously perfused renal parenchyma with arteriovenous shunting and maldistribution of regional cortical-medullary blood flow independent of renal artery flow [47, 64, 66–68]. In conjunction with this, widespread endothelial capillary leak, oxygen utilisation defects at the mitochondria, and hypoxia of nephrons occur which may ultimately result in EAKI [47, 64, 66–68].

### 4.3. Ischaemia-Reperfusion Injury, Haemolysis, and Oxidative Stress

In order to achieve systemic haemodynamic targets and preservation of cardiorespiratory function in both a pre-ECMO setting and a preload-dependant ECMO circuit, certain management strategies are implemented. Frequent use of intravenous (IV) fluid and diuretic therapy, high PEEP strategies (pre-ECMO), regular adjustments in inotrope/vasopressor administration, and variations in ECMO circuit pressures (post-ECMO initiation) commonly occur. In the pre-ECMO setting, these interventions are often prescribed at the expense of renal function as the clinician attempts to delay or prevent the initiation of ECMO. The subsequent delay in the commencement of ECMO with potentially harmful therapy may compound the rapid fluctuations in microvascular blood flow leading to reperfusion EAKI [7, 17, 69]. Alternatively, the restoration of RBF with initiation of ECMO may also contribute to reperfusion EAKI.

Reperfusion injury is likely to be a key pathogenic event in the microcirculatory dysfunction leading to EAKI. It occurs due to inflammatory and oxidative stress-mediated injury, with leukocyte activation and production of reactive oxygen/nitrogen species (RNOS), following restoration of circulation to previously hypoxic cells and subcellular structures [70–72]. Within this process, mitochondrial dysfunction contributes significantly to EAKI [73]. The production of ATP by mitochondria via oxidative phosphorylation drives sodium reabsorption in the kidney tubules [47]. In the setting of multifactorial renal insult, the kidney loses important intrinsic physiological defence mechanisms, which increase the vulnerability of nephrons to oxygen free radicals [74]. ATP production is impaired in the presence of significant oxidative stress due to an increase in mitochondrial protein uncoupling, causing disruption of the mitochondrial membrane potential by the release of hydrogen ions [75]. This process may ultimately contribute to oxidative stress-mediated EAKI.

As mentioned, excess IV fluid therapy before and during ECMO is likely to aggravate this pathophysiological process. Fluid boluses cause recruitment of these hypoxic-shunted renal microcirculatory units and resuscitation of the mitochondria. This “washes/flushes” the hypoxic and acidotic capillary detritus into the circulation, further disrupting oxidative phosphorylation [76]. This also overrides the systemic protective and compensatory vasoconstrictive mechanisms, resulting in maldistribution of oxygen supply [76]. Furthermore, the microcirculatory reperfusion that occurs is ineffective at promoting renal tissue oxygenation due to the poor oxygen carrying capacity of IV fluid [47, 76, 77]. It is clear that the additional inflammation induced by fluid bolus promotes oxidative and inflammatory damage, potentiating tissue demise, and ultimately contributes to EAKI [46, 52]. Novel treatment strategies in humans and animal models of critical illness attempt to prevent and ameliorate microcirculation dysfunction; however none have been performed in ECMO models [50, 76, 78–80]. Also, in view of the negative effects that the conventional use of fluid therapy has on kidney function, future refinements that will be based on microcirculatory variables are currently being considered [76, 81]. At present, it is unclear whether timely initiation of ECMO compared to salvage ECMO and low-circuit pressures compared to high-circuit pressures reduces the effect of reperfusion and oxidative stress-mediated EAKI.

Haemolysis and subhaemolytic damage (leading to erythrocyte deformability), induced by the shear stress of the ECMO circuit, is also likely to contribute to EAKI through oxidative/nitrosative stress [82–88]. Among other variables, the shear stress is likely to be directly related to circuit pressures. The compromise in renal function caused by ECMO-induced haemolysis increases overall mortality in patients on ECMO and this is accentuated by prolongation of the ECC [82, 83, 89–92]. Homeostasis of oxygen, nitric oxide, and RNOS is essential to maintain normal renal signaling, immunity/cellular defence, microvascular function, and cellular respiration [93]. Haemolysis during ECMO disrupts this homeostasis through the effects of elevated levels of free circulating erythrocyte constituents with a relative deficiency of haptoglobin and transferrin [82, 87]. Plasma-free haemoglobin (fHb) and free iron are the main contributors to this process, causing prooxidant/nitrosative damage to the kidney. fHb reacts with hydrogen peroxide and scavenges nitric oxide, which undergoes redox cycling to form toxic free radicals [84, 94, 95]. fHb also converts into methaemoglobin and subsequently precipitates within the renal tubules [92, 96, 97]. Poorly liganded free iron is nephrotoxic as it forms RNOS, such as hydroxyl radicals and superoxide anions, particularly in acidic environments such as the renal tubular urine [94, 98–102]. Additionally, the supraphysiological oxygen content produced by the ECMO circuit leads to the formation of these RNOS in the presence of haemolysis, via the production of superoxide [87, 103–105].

Ultimately, the homeostatic imbalance caused by ECMO-induced haemolysis causes oxidative/nitrosative mediated kidney injury by altering the microvasculature and destruction of the glycocalyx [47, 87, 106]. As mentioned, this promotes leucocyte adhesion, disruption of the endothelial barrier, and glomerular filtration dysfunction [47, 106]. Furthermore, RNOS cause diffuse microcirculatory vasoconstriction, platelet aggregation, lipid peroxidation, and a loss of polarity in renal tubular cells [47, 84, 94, 95, 102, 107]. The damage to important renal cellular and subcellular structures by reactive mediators impairs normal renal function and causes progression to EAKI [47, 106]. The administration of intravenous haptoglobin has shown prophylactic and therapeutic benefits in reducing rates of AKI, highlighting the effect of haemolysis on renal function [82, 108–111]. In animal models, iron chelation with deferoxamine administration is protective against pigment nephropathy [96, 112, 113]. Interventions aimed at reducing the effect of RNOS on kidneys with N-acetylcysteine, iloprost, vitamin C, and dexamethasone and inhibition of inducible NO are promising, predominantly in animal models [53, 114–120]. However, the results of these therapies in more rigorous RCT settings have been disappointing and the effects of these therapies in the setting of ECMO have not yet been investigated. Furthermore, the effect of ECMO circuit pressures on haemolysis is unknown and warrants thorough investigation.

### 4.4. Other

The nonendothelialised ECMO interface and destruction of the glycocalyx may, in isolation or in combination, lead to a hypercoagulable state, which results in the formation of microemboli and microthrombi within the renal vasculature, particularly with VA ECMO [121, 122]. Improvements in ECMO device design (e.g., incorporation of a roller pump with less biocompatible noncoated membrane surfaces) have led to less thrombus formation and an enhanced ability of the circuit to remove larger emboli; however smaller thrombus and emboli still persist [121, 122]. The resultant ischaemia of the renal parenchyma may precipitate AKI while on ECMO and result in EAKI. Novel treatment strategies that prevent thrombosis partly by preserving glycocalyx integrity, such as soluble thrombomodulin (sTM) and activated protein C, are being investigated in the setting of sepsis-associated AKI; however their efficacy in EAKI is unknown [48, 50].

Patients supported on VV ECMO with respiratory failure and receiving lung protective ventilation have often had prolonged hypercapnia. Despite correction by ECMO, this induces significantly altered haemodynamics and RBF [124]. This can lead to acidosis, hypoxia, poor pulmonary compliance, and pulmonary hypertension, which can progress to* cor pulmonale*, shock, and organ dysfunction. The primary disease process in conjunction with ongoing lung injury and altered haemodynamics induced by ventilation may further exacerbate organ injury and increase the risk of EAKI [125]. To this end, timely initiation of ECMO may mitigate pre-ECMO risk factors for organ dysfunction and EAKI. Alternatively, raised intrathoracic pressure secondary to high positive-end expiratory pressure (PEEP) on ECMO may induce renal hypoperfusion and impair the kidneys excretory function [126, 127]. However, high PEEP in conjunction “ultralow” tidal volumes may actually help to reduce the overall intrathoracic pressure and ameliorate the potential negative effects on kidney function.

As mentioned, the centrifugal ECMO pumps are preload dependant and deficient in the Frank-Starling mechanism; thus maintenance and bolus IV fluid administration are necessary to maintain adequate ECMO flow and minimise “suckdown,” particularly in the immediate days following ECMO initiation [128]. However, a positive fluid balance on day 3 of ECMO correlates with higher mortality [128]. Conversely, the subsequent use of diuretic therapy in order to achieve neutral fluid balance, minimise extravascular lung water, and improve gas exchange and pulmonary compliance may also further exacerbate EAKI [129].

Despite the pulsatility differences between VV and VA ECMO, provided that ECMO circuit flow rates are in the range of premorbid cardiac output, there is a lack of evidence to suggest that a nonpulseless system increases the risk of EAKI. Initiation of peripheral VA ECMO can lead to profound reperfusion and hyperaemia, which may exacerbate ischaemia-reperfusion injury. On the contrary, among other mechanisms, overall renal perfusion may improve as a result of the reduced venous pressure induced by the negative drainage pressure in the IVC.

An increased duration of support with ECC is an independent risk factor causing an increased incidence of AKI and subsequently higher morbidity and mortality [130]. This highlights the likelihood of an AKI that is induced by an extracorporeal circuit. As a result of the extended duration of therapy and the presence of significant acute and preexisting underlying disease of patients on ECMO, these factors are likely to cause greater consequences. This may be reflected by the high incidence of AKI with ECMO. On the other hand, management strategies that are implemented to prevent or delay initiation of ECMO in a critically ill patient may also be the predominant factor leading to high rates of EAKI. EAKI is not yet established within the literature, warranting further consideration and subsequent investigation into the “causation versus association” relationship to determine whether ECMO is a “friend or foe” to renal function.

## 5. Methods for Investigating ECMO-Associated Kidney Injury

A number of tools can detect and define the consequences that the various pathophysiological processes associated with EAKI have on renal function. Methods for determining AKI can be categorised into broad groups: physiological, biochemical, radiological, histological/immunohistochemical, and biomarker evaluation (Table 3).

### 5.1. Physiological and Biochemical

In current clinical practice, the use of broad physiological end-points (i.e., urine output) and biochemical (i.e., serum creatinine, blood/urine urea nitrogen, and blood/urine electrolytes) are used. These indices are synthesised using RIFLE, AKIN, and calculations to determine the fractional excretion of sodium (FeNa) and urea (FeUrea) [28, 29, 131]. Calculation of GFR is extremely important in the evaluation of kidney function and can be achieved using a variety of methods. The use of gold standard filtration markers, such as inulin and iohexol, are limited in clinical practice [131]. However new techniques for determining GFR are emerging which may be beneficial in the setting of EAKI. These include the use of a transcutaneous remote real-time analysis of FITC-sinistrin excretion and a point-of-care bedside fluorescence-based measured GFR (mGFR) assay [131]. Other biochemical markers such as fHb, plasma-free myoglobin, red cell distribution width (RDW), bilirubin, haptoglobin, plasma-free iron, hepcidin, plasma/urine albumin, and components of the renin-angiotensin aldosterone system (RAAS) may help to determine the risk and nature of EAKI [82, 95]. Circulating levels of syndecan-1, endocan, and selectins may be useful markers of glycocalyx degradation [48, 132]. Hemeprotein release and lipid peroxidation can be determined by the presence of elevated plasma/urine F_2_-isoprostanes and isofurans, which correlate with oxidative stress-mediated kidney damage [95].

Advanced physiological parameters can be obtained through invasive procedures. Renal and pulmonary artery transit-time flow probes with oximetry capability may facilitate a deeper understanding of EAKI. Moreover, they may allow accurate measurement of macro/microvascular flow, renal cortical and medullary tissue perfusion, and renal function throughout the entire course of ECMO in animal models [57, 133]. This can be achieved through an ultrasound-guided or surgically inserted renal artery catheter and subsequent assessment of renal blood flow, renal vascular conductance, and tissue oxygenation [57, 133]. Assessment of renal microcirculatory function and tissue oxygenation with near infrared spectroscopy (NIR), urine oxygen tension, tissue microdialysis, mitochondrial function analysis, and respiratory electron chain transport using Orboros may also provide investigative benefit [131].

### 5.2. Imaging

Imaging studies may be utilised to define the presence and severity of AKI in patients supported by ECMO. Renal ultrasound ± Doppler is the most commonly utilised imaging modality for the assessment of patients with AKI [134]. Although there is no current data on its use in ECMO, it can be easily used in patients supported by ECMO and its utility should be investigated. Assessment of renal size, echogenicity, Doppler flow, and resistive index (RI) may help to predict, distinguish between causes, and provide prognostic information for AKI in patients on ECMO [57, 134–136]. However, limitations exist as RI can be influenced by a number of extrarenal factors such as heart rate and peripheral vascular resistance and therefore lacks specificity for AKI [137].

Contrast enhanced ultrasonography (CEU) uses contrast agents composed of microbubbles with an injectable gas in a supportive shell of phospholipids or proteins [138]. CEU can identify the intrinsic spatial and temporal heterogeneity of renal parenchymal perfusion [131, 139]. It has been shown to do this with a greater degree of accuracy compared to other imaging modalities, including magnetic resonance (MR) and computerised tomography (CT) [140]. Along with laser Doppler flowmetry, it is an ideal imaging modality for studying the microcirculation of organ blood flow/velocity patterns [141, 142]. Unlike other modalities CEU is safe, free of disruptive haemodynamic effects, and easy to utilise in patients on ECMO; however investigation into the destructive effects of the circuit interface on the concentration and utility of the contrast microspheres is warranted [143–145]. Due to logistical and incompatibility issues related to the ECMO circuit, recent advancements in other imaging modalities cannot be utilised to investigate EAKI. As such radiological assessment of renal medullary and cortical oxygenation in response to haemodynamic variables on ECMO is relatively limited.

### 5.3. Histological and Immunohistochemical

Renal biopsies in order to assess the presence, nature, and extent of AKI are not performed in patients on ECMO due to an unfavourable risk : benefit ratio; however, its use may be helpful in experimental animal models. Important pathophysiological factors may be identifiable through histological and immunohistochemical assessment of nephrons. Conventional staining methods combined with various immunohistochemical staining, apoptosis quantification, and gene expression for glycocalyx disruption and major controllers of vessel tone may delineate the cause and extent of EAKI. Among other methods, transmission electron microscopy (TEM) with ruthenium red, ferritin, lanthanum, and cupromeronic blue staining may help to specifically identify renal glycocalyx disruption [146]. Additionally, histological analysis with immunostaining of pimonidazole and the Pd phosphorescence technique can identify hypoxic areas of renal tissue, potentially highlighting an underlying heterogenous hypoxic nature of EAKI [147, 148].

## 6. Potential Role of Biomarkers in ECMO-Associated Kidney Injury 

Whilst the role of novel biomarkers in AKI has shown promise they have been underutilised for patients on ECMO. In 2005, the Council of the American Society of Nephrology recommended that the highest research priority be the standardization and discovery of new biomarkers of AKI [149]. Following this, numerous publications outlining the use of many new biomarkers have emerged; however very few have been studied in patients on ECMO (Table 3: novel biomarkers) [150]. A number of biomarker-specific limitations and unresolved concerns have prevented their widespread use in clinical practice; however refinements are ongoing and an increase in clinical integration is likely to occur [151, 152].

Neutrophil gelatinase-associated lipocalin (NGAL) and cystatin C (CystC) have the greatest depth of research for the detection of AKI. Other promising biomarkers include kidney injury molecule-1 (KIM-1), renal-liver type fatty acid binding protein (L-FABP), insulin-like growth factor-binding protein 7 (IGFBP7), and tissue inhibitor of metalloproteinases-2 (TIMP-2). Combining these biomarkers may collectively improve the sensitivity and specificity of detecting AKI in patients supported by ECMO [153]. Novel biomarkers for AKI have been extensively assessed in the critical care setting and in patients supported by an ECC; however none have studied their utility in adults on ECMO [154–159]. Most of these markers can be easily measured in urine and plasma and their concentration increases with duration and severity of acute tubular injury [154, 155, 160–163]. These biomarkers have demonstrated benefits in regard to early detection, need for RRT, recovery of kidney function, and prediction of prognosis of AKI in a variety of clinical settings [37, 44, 105, 113, 150, 157, 159, 163–179]. Based on the incidence and clinical sequelae of EAKI, the utility of novel biomarkers in this cohort may be beneficial, particularly with regard to delineating major renal insult, and should be investigated.

Earlier detection of kidney injury using new biomarkers has led to the recognition of a subgroup of patients with “subclinical” or “incipient” AKI [161, 180–182]. “Subclinical AKI” is a term used to describe a state where tubular damage without glomerular function loss has occurred [180]. Moreover, it refers to when the RIFLE criteria are negative, but elevated levels of new biomarkers are present [180]. This subgroup is found to be associated with worse clinical outcomes [161, 180, 181]. Clinical use of “subclinical AKI” is not yet definitive as the specific clinical applications of biomarkers are currently undefined [152]. Nevertheless, investigating AKI with biomarkers has improved our understanding of pathophysiological processes and refined our approach to treatment strategies [151]. In the ECMO setting, biomarkers may be a vital tool for understanding whether initiation of ECMO is protective or destructive for renal function. Ultimately, earlier detection with biomarkers may enable early evidence-based risk stratification for diagnostic evaluation, monitoring AKI, and initiating/discontinuing “renal protective” interventions for patients on ECMO [183].

## 7. Experimental Models to Investigate ECMO-Associated Kidney Injury

The multitude of complex, interrelated variables present in patients on ECMO are difficult to control in a study setting. As a result, clinical research in patients on ECMO is challenging. Simulated ECC and* in vivo* animal models provide a valuable means to undertake detailed, systematic research into complex clinical scenarios and pathophysiological changes associated with ECMO [184]. Furthermore,* in vivo *models with large animal subjects may help to delineate and define the relative risk of EAKI. An* in vivo* ovine model has many advantages [10, 184]. Firstly, the overall size and weight are similar to humans. Secondly, sheep have similar renal anatomical structure, haemodynamic physiology, and microcirculatory function, which can be investigated with advanced methods/tools that are clinically applicable to human subjects [10, 145, 185–187]. Finally, ovine models have been used extensively throughout the literature for other human conditions [188–190].

An* in vivo*, ovine model of VV ECMO may successfully mimic the severity of patient illness and reproduce key haemodynamic and immunological aberrations. It can also serve as its own control, mimic histological findings in relevant organs, and provide a means to gain insight into factors contributing to interpatient variability [184]. Ultimately, an* in vivo* model will allow a thorough investigation into the renal response to various clinical conditions. Translational clinical investigations into EAKI will lead to a more accurate assessment of the relative contribution of ECMO circuit factors on renal function. Moreover, implementing novel renal monitoring modalities based on relevant pathophysiology will provide novel strategies for preventing/treating EAKI.

## 8. Conclusion

There are limited data within the literature exploring EAKI. Furthermore, there are no studies using RIFLE/AKIN in conjunction with advanced methods for investigating AKI in patients supported by ECMO. The high incidence rate of AKI in patients supported on ECMO and the subsequent mortality that emerges as a result of AKI is unacceptable. It is likely that EAKI exists as a dependent entity; however it is equally possible that initiation of ECMO serves to protect renal function. Understanding the patient-device interface and its role in the progression of normal renal function to AKI is imperative. This, in conjunction with earlier detection with the use of novel investigative methods, is fundamental step towards reducing EAKI. These issues can be investigated using an* in vivo *animal model with the goal to improve the understanding of the changes observed during ECMO and, subsequently, optimise the process. A better understanding of this will help to define optimal renal support for patients during therapy, ultimately reducing the risks associated with ECMO and improve patient survival.

## Figures and Tables

**Table 1:** The AKIN and RIFLE criteria for identification and staging of AKI.

AKIN criteria^*∗*^			RIFLE criteria^∧^		
Stage	Creatinine	Urine output	Stage	Creatinine or GFR	Urine output
1	Increase of >0.3 mg/dL or 0.5–2 times (baseline)	<0.5 mg/kg/hr for >6 hr	Risk	sCr increase ×1.5 (baseline) or GFR decrease >25%	<0.5 mg/kg/hr for >6 hr
2	2-3 times (baseline)	Anuria for 12 hr or <0.5 mg/kg/hr for >12 hr	Injury	sCr increase ×2.0 (baseline) or GFR decrease >50%	<0.5 mg/kg/hr for 12 hr
3	RRT or level of 4.0 mg/dL with an acute increase of 0.5 mg/dL	<0.3 mg/kg/hr for 24 hr	Failure	sCr increase ×3.0 (baseline) or GFR decrease >75% or 4.0 mg/dL with an acute increase of 0.5 mg/dL	<0.5 mg/kg/hr for 12 hr
			Loss	Persistent AKI (loss of renal function >4 weeks)	
			ESKD	End stage kidney disease >3 months	

^*∗*^Renal assessment time window up to 48 hours.

^∧^Renal assessment time window up to 7 days.

**Table 2:** Kidney injury-specific independent predictors of mortality during various stages of ECMO treatment.

Author	Journal	Year	Period	Number of patients	Relation to ECMO	AKI-specific independent predictors
Lin et al. [11]	Ann Thorac Surg	2007	2002–2005	78	On ECMO	RIFLE
Yan et al. [31]	Euro J Cardiothoracic Surg	2010	2004–2008	67	On ECMO	Class failure (RIFLE), stage 3 (AKIN)
Chen et al. [18]	Ann Thorac Surg	2011	2002–2008	102	Post-ECMO (48 hours)	AKIN (48 hours)
Chang et al. [123]	PLoS One	2012	2006–2010	119	Post-ECMO	Day 2 urine output after ECMO
Hsiao et al. [16]	Ann Thorac Surg	2014	2006–2011	81	Concomitant ARDS	Urine output

**Table 3:** Potential methods for investigating EAKI.

Physiological	Biochemical
Urine output (UO) Blood pressure (BP), cardiac output (CO), and central venous pressure (CVP) Renal blood flow (RBF), renal vascular conductance (RVC) Renal artery transit-time flow probes with oximetry Urine oxygen tension	Serum/urine creatinine (sCr, uCr), blood urea nitrogen (BUN), and serum potassium (K+) Fractional excretion of sodium/urea (FeNa/FeUrea) Creatinine clearance (CC), RIFLE and AKIN Red cell distribution width (RDW), bilirubin, haptoglobin, hepcidin, and plasma/urine albumin Cockcroft-Gault equation (eGFR), fluorescence-based measured GFR (mGFR) assay, and FITC-sinistrin excretion

Radiological	Histological

Renal ultrasound + Doppler Contrast-enhanced ultrasound (CEU) Near infrared spectroscopy (NIR)	General—hematoxylin and eosin stain (H&E) Fibrosis/collagen—Masson's trichome Protein—periodic acid-Schiff (PAS) reagent Lipofuscin—Schmorl's stain, iron—Perl's stain, and other: deposits, casts, and necrosis

Immunohistochemistry/other	Novel biomarkers (serum/urine)

Neutrophils, macrophages—anti-CD68, Myofibroblasts—anti-*α*-smooth muscle actin (SMA) Apoptosis quantification—cleaved caspase-3 Nitric oxide synthase, pimonidazole, and Pd phosphorescence Syndecan-1, endocan, and selectin Transmission electron microscopy with ruthenium red, ferritin, lanthanum, and cupromeronic blue stain	Neutrophil gelatinase-associated lipocalin (NGAL), Cystatin C (CystC), kidney injury molecule (KIM-1), IL-6, and IL-18 N-acetyl-glucosaminidase (NAG), renal-liver type fatty acid binding protein (L-FABP) Insulin-like growth factor-binding protein 7 (IGFBP7), and tissue inhibitor of metalloproteinases-2 (TIMP-2) Syndecan-1, endocan, and selectin F_2_-isoprostanes and isofurans
